# Factors Associated with Treatment Satisfaction Among People Living with HIV in Japan and Other Selected Countries: Examination of the Intertwined Roles of Medication, Patient, and Provider Characteristics

**DOI:** 10.1007/s10461-021-03515-2

**Published:** 2021-12-06

**Authors:** Ichiro Koga, Rumi Wakatabe, Noriko Okamoto, Asuka Sasai, Keita Kambara, Andres Maldonado, Patricia de los Rios, Chinyere Okoli

**Affiliations:** 1 ViiV Healthcare, Tokyo, Japan; 2 Japanese Network of People Living With HIV/AIDS (JANP Plus), Tokyo, Japan; 3 ViiV Healthcare, Wavre, Belgium; 4 ViiV Healthcare, Laval, QC Canada; 5 ViiV Healthcare, Brentford, UK

**Keywords:** Treatment satisfaction, Quality of life, Antiretrovirals, Medication, HIV

## Abstract

We examined satisfaction and perceived challenges with antiretroviral therapy (ART) among people living with HIV (PLHIV) in Japan vs three other Asian countries (China, Taiwan, South Korea), and 21 non-Asian countries, using data from the 2019 Positive Perspectives Study (pooled sample size from all 25 countries = 2389). Participants in other Asian countries were more likely than those in Japan to report they missed ART ≥ 1 time in the past month because they were depressed/overwhelmed (57.4%[89/155] vs 32.0%[24/75]), had privacy concerns (56.8%[88/155] vs 30.7%[23/75]), were concerned about the potential long-term negative impacts of ART (46.5%[72/155] vs 26.7%[20/75]), or just wanted to forget about HIV (45.8%[71/155] vs 22.7%[17/75]). ART satisfaction however did not differ significantly between surveyed PLHIV in Japan (54.7%[41/75]) vs those in other Asian countries (47.7%[74/155]). The percentage who felt that daily ART dosing limited their lives was 36.0%[27/75] among participants from Japan, 48.4%[75/155] among participants from other Asian countries, and 27.3%[589/2159] among those from non-Asian countries. Within a structural equation model using pooled data from all 25 countries, positive correlations were seen between ART satisfaction and “provider engagement” (β = 0.35), high perceived control over ART dosing schedule (β = 0.28), and the belief that ART prevents HIV transmission (β = 0.16). Conversely, negative correlations were seen between ART satisfaction and experience of ART side-effects (β = − 0.24), high “ART anxiety” (β = − 0.20); and being on multi-tablet regimens (β = − 0.13). Those ART-satisfied reported higher self-rated health and greater ART adherence. These findings underscore the need for patient-centered care to enhance treatment satisfaction and improve ART adherence.

## Introduction

HIV has changed. People living with HIV (PLHIV) are now able to live longer, fuller lives, experience better quality care, and expect not just to survive, but to thrive [1–4]. The important task of improving the person-centeredness of HIV care falls on no one person but rather requires an all-hands-on-deck approach—PLHIV, healthcare providers (HCPs), regulators, drug manufacturers, and other members of the healthcare ecosystem [5]. The benefits are however enormous not only for the individual patient but the entire health system and the general population. According to the triple aim framework developed by the Institute for Healthcare Improvement [6], the performance of health systems can only be optimized by simultaneously improving the patient experience of care, improving the health of populations, and reducing the per capita cost of healthcare.

It is easy to describe the benefits of positive patient experience of care but harder to quantify “experience of care” as a single summary metric because it is inherently time-varying, subjective, and multidimensional. For example, patients may have different levels of satisfaction with their main provider, vs other members of the health team, vs their antiretroviral therapy (ART). Within pooled data from the 2019 Positive Perspectives Study, 32.7% of PLHIV felt their main HCP did not prioritize their needs/preferences while 61.2% and 39.1% felt there was room for improving their HIV overall management and HIV medications, respectively [7]. PLHIV may be dissatisfied with their treatment even if they are virally suppressed or report good overall health [8], underscoring the need for ongoing patient-provider communication. Yet, many PLHIV hold back from such discussions for fear of being labelled a difficult patient [7]. Furthermore, face-to-face patient-provider engagements may have been limited in some areas because of the ongoing COVID-19 pandemic.

Experiences associated with daily oral ART are likely the most consequential to PLHIV because of the sheer frequency of intake [9]; it is therefore the focus of this paper. There is a paucity of data on how subjective experiences with ART are influenced by the characteristics of the medication, the clinical contexts in which it is offered, and the psychographic characteristics of the person taking the medication. To fill this gap in knowledge, this study used a structural equation model (SEM) to examine these multiple, inter-related associations among a pooled sample of 2389 participants in 25 countries. In view of the rising HIV incidence rate in Japan compared to most other countries as well as other unique cultural and contextual factors [10–12], we further performed descriptive analyses to contrast ART satisfaction and perceived treatment challenges in Japan vs other Asian countries (China, Taiwan, and South Korea), as well as non-Asian countries (Argentina, Australia, Austria, Belgium, Brazil, Canada, Chile, France, Germany, Italy, Mexico, Netherlands, Poland, Portugal, Ireland, Russia, South Africa, Spain, Switzerland, UK, and USA). The rationale for these comparisons was to determine whether treatment experiences reported by PLHIV in Japan were unique relative to regional or global patterns. Specific questions explored as part of this study were: (1) What treatment challenges do PLHIV face that might influence perceived ART satisfaction and how do these differ among PLHIV in Japan vs other Asian countries and non-Asian countries? (2) How do various treatment experiences influence ART satisfaction, and what is the impact on indicators of quality of life? (3) What improvements in ART are considered most important to PLHIV, comparing those in Japan, vs other Asian countries and non-Asian countries?

## Methods

### Data Source

The 2019 Positive Perspectives Study was conducted in 25 countries among 2389 PLHIV aged 18–84 years [7–9, 13–16]. All participants were on ART at the time of the survey and provided informed consent. Ethical review was provided by the Pearl Institutional Review Board (no. 18-080622). In addition, specific approval for South Africa was obtained from the Sefako Makgatho Research Ethics Committee (no. SMUREC/M/223/2019). The Positive Perspectives survey was sponsored by ViiV Healthcare but fielded independently by Ipsos Healthcare. To reduce potential bias in completed responses, there was double blinding: ViiV Healthcare was not identified to participants as the study sponsor; likewise, participants’ identities were not revealed to ViiV Healthcare.

### Measures

#### Experiences with ART

ART satisfaction was assessed as follows: “Overall, how satisfied are you with your current HIV medication?” Response options were 1-“Very dissatisfied”, 2-“Dissatisfied”, 3-“Neither satisfied nor dissatisfied”, 4-Satisfied”, and 5-“Very satisfied”. For descriptive analyses, scores of ≥ 4 were classified as a positive indication of satisfaction.

ART formulation was dichotomized as ≤ 1 tablet/day (henceforth, single tablet regimen or STR) vs > 1 tablet/day (henceforth, multi-tablet regimens or MTR). We further reviewed the free-text responses provided by those answering “Other” and classified those accordingly as STR (e.g., “1 tablet 5 days a week”) or MTR (e.g., “Liquid meds twice a day”). Those answering “Other” without a specified frequency were set to missing to avoid misclassification (11/2389 individuals).

Participants indicated what aspects of their ART they worried about, including: “how taking HIV medicines for many years will impact my body”; “having to take more and more medicines as I get older”; “how my HIV medicines will affect other medications/drugs/pills I take”; “that the long-term impact of HIV medicines is unknown”; “how my HIV medicines will impact my overall health and wellbeing”; “that I will run out of HIV treatment options in the future”; and “the long-term side effects of my HIV medication”. Attitudes towards daily oral dosing and treatment advances were further measured on a 5-point ordinal scale: “Taking my pill(s) every day reassures me that my HIV is being kept under control”; “Having to remember to take my HIV medication every day causes me stress or anxiety”; “Taking my HIV medication limits my day-to-day life”; “I have no problem managing the pill(s) I need to take each day for my HIV”; “Taking pills for HIV every day is a daily reminder of HIV in my life”; “Taking pills for HIV every day is a link to some bad memories from my past”; “I worry about forgetting to take my daily HIV medication or taking it later than planned”; “I worry that having to take pills every day means a greater chance of revealing my HIV status to others”; “As long as my HIV stays suppressed, I would prefer not having to take HIV medication every day”, and “I believe that future advances in HIV treatment will improve my overall health and wellbeing”. Scores of ≥ 4 were classified in descriptive analyses as an affirmative response. Reasons for missing ART ≥ 1 time within the past month were assessed; suboptimal adherence was defined as a report of at least one reason for missing ART ≥ 5 times within the past month [13].

As part of the survey, a maximum diffusion experiment was conducted where participants were shown a set of statements concerning potential improvements to HIV medicines and asked to rank the most and least important to them [8]. The seven improvements ranked were: “Smaller pills”; “Fewer side effects”; “Reduced long-term impact on my body”; “Less chance of affecting other medicines/drugs/pills I take”; “No food restrictions or requirements”; “Less HIV medicine each day but just as effective”; and “Longer-lasting medicine so I don’t have to take it every day (for example, a monthly injection administered by a doctor/nurse)”.

#### Experiences with HIV Care Providers

Communication with providers was assessed with the following indicators measured on a 5-point ordinal scale: “I am given enough information to be involved in making choices about my HIV treatment”; “I feel I understand enough about my HIV treatment”; “My provider seeks my views about treatment before prescribing an HIV medication”; “My provider asks me if I have any concerns about the HIV medication I am currently taking”; “My provider tells me about new HIV treatment options that become available”; “I would like to be more involved when it comes to decisions about my HIV treatment”; “My provider asks me frequently about any side effects I might be experiencing with my HIV treatment”; “My provider has told me about “undetectable = untransmittable” (U = U)”. Scores of ≥ 4 were classified in descriptive analyses as an affirmative response. Perceived comfort discussing specific issues with HCPs was also assessed, as well as perceived barriers to engaging in such discussions.

#### Self-rated Health

Self-rated health was measured separately across four domains, each on a 5-point ordinal scale: physical, mental, sexual, and overall health. Scores of ≥ 4 were classified in descriptive analyses as optimal health.

#### Other Demographic, Social, and Clinical Characteristics

These included age, gender, sexual orientation, employment, and duration of HIV. Participants were also asked reasons for not sharing their HIV status with others.

### Analyses

#### Comparison of ART Satisfaction and Treatment Challenges Among Participants in Japan, Other Asian Countries, and Non-Asian Countries

We contrasted prevalence of emotional and psychosocial treatment challenges among participants in Japan (n = 75), other Asian countries (n = 155), and other non-Asian countries combined (n = 2159). Within- and between-group comparisons were done in R version 3.6.1 with χ^2^ tests at p < 0.05. All percentages were computed based on dichotomized variables (coded as 0 = no, 1 = yes).

#### SEM of Correlates of ART Satisfaction Within the Pooled Sample

**Fig. 1:**
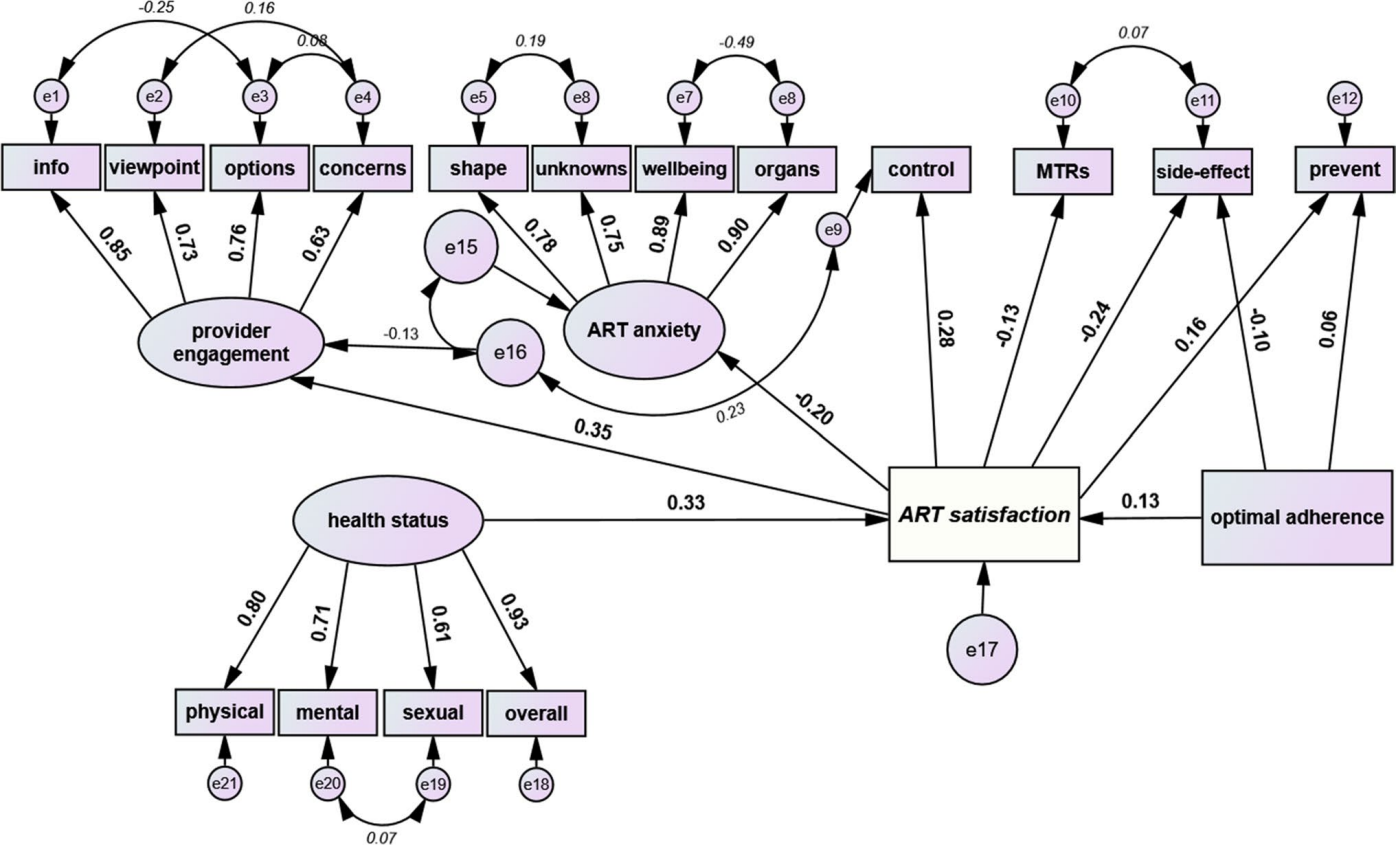
Final structural model assessing the relationship between ART satisfaction^a^ and “provider engagement”^b^, “ART anxiety”^c^, perceived control over ART dosing schedule^d^, use of multi-tablet regimens (MTRs), experience of ART side effects^e^, and belief that ART prevents HIV transmission^f^, Positive Perspectives Study, 2019 (n = 2389). *ART* antiretroviral therapy; *MTR* multi-tablet regimens. Standardized estimates in bold are correlations and factor loadings (single arrows). Those *italicized* are correlations between error terms (double arrows). ^a^ART satisfaction was assessed as follows: “Overall, how satisfied are you with your current HIV medication?” Responses were measured on an ordinal scale from 1 to 5. ^b^*Info* = “I am given enough information to be involved in making choices about my HIV treatment”; “I feel I understand enough about my HIV treatment”; *viewpoint* = “My provider seeks my views about treatment before prescribing an HIV medication”; *options* = “My provider tells me about new HIV treatment options that become available”; *concerns* = “My provider asks me if I have any concerns about the HIV medication I am currently taking”. ^*c*^*shape* = “I worry how taking HIV medicines for many years will impact my body/body shape”; *unknowns* = “I worry that the long-term impact of HIV medicines is unknown”; *wellbeing* = “I worry how my HIV medicines will impact my overall health and wellbeing”; “I worry about the long-term side effects of my HIV medication” (e.g., problems with bones, kidneys, liver). One covariance path was present in the model but not displayed in the figure above for the sake of simplicity and clarity; the double arrow connects e15 with e11 (covariance = 0.32). ^d^*Perceived control* over ART dosing schedule (variable with error term e9) was measured by extent of agreement with the statement “I have no problem managing the pill(s) I need to take each day for my HIV”. ^e^*Self-reported side effects* experience *(variable with error term e11)* was measured by extent of agreement with the statement “My current HIV medication gives me side effects”. ^f^The perception ART does *prevent* HIV transmission *(variable with error term e12)* was measured by extent of agreement with the statement “My HIV medication prevents me from passing on HIV to others”

SEM is a robust multivariable technique particularly suited for examining attitudinal and behavioural outcomes such as treatment satisfaction which reflect complex, multiple, inter-related and multidirectional relationships. In our SEM, we first measured out the variables of interest (i.e., the measurement model), then we determined the structure/relationship of one variable to the other (i.e., the structural model). The measurement model involved creating unseen variables (i.e., latent variables, formulated from a theoretical and statistical framework, and represented by oval shapes) from seen variables (i.e., observed variables from the dataset, represented by rectangular shapes). The reason for specifying latent variables is that they capture the construct of interest in greater depth and breadth as they summarize information from several observed variables, each of which may only tell part of the story on their own. In Fig. 1, there are three latent variables, “health status”, “provider engagement” and “ART anxiety”, each of which has four explanatory variables; single-headed arrows lead from the latent variable to the observed variables to signify a measurement model. The numbers beside each arrow in the measurement model (factor loadings) tell the proportion of the variation in the individual observed items that is explained by the latent factor (range 0 to 1, the bigger the better). The left-over variation not explained is termed the measurement error, the error variables are represented by small circles. A connecting double-headed arrow between two error variables means there is an expectation that the two change in tandem. For the structural part of our SEM which sought to measure the strength of association between indicated variables, single-headed arrows leading away from a latent or observed variable indicate an outcome variable, while single-headed arrows leading to a latent or observed variable indicate an explanatory variable. The strength of the specified relationships is presented as correlations (β), which as standardized values, can only range from − 1 to + 1; larger absolute values signal stronger associations. Negative β values indicate negative correlations (mean value of the one variable decreases as that of the other increases) and positive values indicate positive correlations (mean value of the one variable increases as that of the other increases too). Accompanying confidence intervals for β that go from negative for the lower limit, to positive for the upper limit (i.e., includes 0) indicate statistically nonsignificant results.

We conceptualized two overlapping theoretical frameworks for exploring determinants of ART satisfaction in our SEM. The first was the pre- and post-administration framework, which posited that ART satisfaction could be influenced by certain conditions before (e.g., formulation of the medication) as well as after intake of the medication (e.g., ensuing side effects). The second was the control-reward framework, which posited that the extent of ART satisfaction was, on the one hand, a function of the extent PLHIV felt in control of their medication (high control if they felt in control of their dosing schedule in their day-to-day life; low control if they felt their dosing schedule controlled their day-to-day lives), as well as perceived benefits from the medication on the other hand (e.g., the belief that ART prevents HIV transmission).

The a priori specified SEM in Stata Version 14.0 and Amos Version 26 explored the following: (1) High “provider engagement”, high perceived control over ART dosing schedule, and high levels of subjective belief regarding benefits of treatment would be positively related to ART satisfaction. (2) Conversely, high levels of “ART anxiety”, side effects, and MTRs would be negatively related to ART satisfaction. (3) “ART satisfaction” would in turn be positively related to optimal adherence and to good “health status”.

Subsequently, confirmatory factor analysis was performed using SEM to test our a priori hypotheses; adjustments were made to the specified general model by removing non-significant pathways and correlating error terms based on modification indices. Adequacy of model fit was assessed using Tucker Lewis Index (TLI, > 0.9); Comparative fit Index (CFI, > 0.9) and Root Mean Square Error of Approximation (RMSEA, ≤ 0.06) [17–21]. TLI and CFI measure comparative fit (i.e., how well does the model fit against a null or independence model?) - the bigger the better. RMSEA measures absolute fit (i.e., how well does the model fit against a perfect model?) - the smaller the better. All ordinal variables were analyzed as such within SEM (coded as 1 through 5, lowest through highest).

#### Maximum Diffusion Analysis of What ART Improvements PLHIV Value the Most

Using R Version 3.6.1, we calculated the weighted probabilities that each individual attribute was ranked in the 1st, 2nd, …7th position [8]. Across all attributes, we also calculated the “preference shares” for each position. The percentage who ranked each attribute as first or second position was calculated and compared for Japan (n = 62), other Asian countries (n = 133), and non-Asian countries (n = 1963). The analytical sample sizes for the maximum diffusion experiment are less than the full sample sizes within descriptive analyses as not all respondents participated in the experiment, either because they used paper-and-pencil questionnaires, or they chose to skip that part of the survey.

## Results

### Characteristics of the Study Population

Socio-demographic characteristics of participants in Japan, other Asian countries, and non-Asian countries are presented in Table 1. Within Japan, mean (SD) age and duration of HIV were 38.1 (12.1) and 8.0 (6.9) years, respectively; 66.7%[50/75] were men, 76.0%[57/75] were aged < 50 years, and 58.7%[44/75] lived in metropolitan areas. Furthermore, 24.0%[18/75] of Japanese participants were recently diagnosed with HIV during 2017–2019 and 57.3%[43/75] reported having ≥ one non-HIV comorbidity. The most common comorbidities reported among participants in Japan were mental health disorders (14.7%[11/75]), insomnia or other sleep disorder (13.3%[10/75]), anemia (12.0%[9/75]), hypertension (10.7%[8/75]), and heart disease (9.3%[7/75]); prevalence of self-rated optimal heath, by domain, was: physical, 52.0%[39/75]; mental, 46.7%[35/75]; sexual, 49.3%[37/75] and overall, 53.3%[40/75].

**Table 1:** Percentage who reported satisfaction with their HIV medication among people living with HIV in Japan, other Asian countries, and non-Asian countries combined, Positive Perspectives Study, 2019

Indicator	Categories	Composition of the study population, % (N)			Percentage satisfied with their HIV medication, %^c^		
		Japan	Other Asian countries^a^	Non-Asian countries^b^	Japan	Other Asian countries	Non-Asian countries
Total	Overall	100.0 (75)	100.0 (155)	100.0 (2159)	54.7	47.7	71.4
Age	< 50	76.0 (57)	79.4 (123)	69.9 (1510)	49.1	50.4	69.6
	50+	24.0 (18)	20.7 (32)	30.1 (649)	72.2	37.5	75.7
Gender	Men	66.7 (50)	68.4 (106)	68.0 (1467)	62.0	55.7	73.3
	Other gender	1.3 (1)	0.0 (0)	3.2 (69)	–^d^	–^d^	63.8
	Women	32.0 (24)	31.6 (49)	28.9 (623)	41.7	30.6	67.9
Year of HIV diagnosis	2017 to 2019	24.0 (18)	29.0 (45)	22.5 (485)	50.0	46.7	66.2
	2010 to 2016	46.7 (35)	54.2 (84)	36.8 (794)	51.4	45.2	73.7
	Pre-2010	29.3 (22)	16.8 (26)	40.8 (880)	63.6	57.7	72.3
Sexual orientation	Heterosexual	36.0 (27)	53.6 (83)	40.5 (874)	40.7	37.3	63.6
	Homosexual	37.3 (28)	34.8 (54)	46.9 (1012)	71.4	70.4	77.8
	Bisexual/asexual/other	26.7 (20)	11.6 (18)	12.6 (273)	50.0	27.8	72.9
ART pill formulation	Single tablet regimen	20.3 (15)	42.2 (65)	49.8 (1071)	73.3	60.0	74.7
	Multi-tablet regimen	79.7 (59)	57.8 (89)	50.2 (1079)	50.8	39.3	68.3
Disguising HIV pills in past 6 months	No	36.0 (27)	16.8 (26)	44.1 (953)	70.4	57.7	78.5
	Yes	64.0 (48)	83.2 (129)	55.9 (1206)	45.8	45.7	65.8
Perception daily oral ART limits life	No	64.0 (48)	51.6 (80)	72.7 (1570)	60.4	65.0	75.7
	Yes	36.0 (27)	48.4 (75)	27.3 (589)	44.4	29.3	59.9
Perception daily oral dosing is reminder of HIV	No	57.3 (43)	40.7 (63)	41.2 (889)	58.1	68.3	73.3
	Yes	42.7 (32)	59.4 (92)	58.8 (1270)	50.0	33.7	70.1
Experience side effects from current ART	No	60.0 (45)	51.0 (79)	56.7 (1224)	60.0	55.7	76.6
	Yes	40.0 (30)	49.0 (76)	43.3 (935)	46.7	39.5	64.6
Believe ART prevents HIV transmission	No	41.3 (31)	32.9 (51)	24.7 (534)	45.2	31.4	59.7
	Yes	58.7 (44)	67.1 (104)	75.3 (1625)	61.4	55.8	75.3
Perceive HCP meets their needs and priorities	No	37.3 (28)	45.2 (70)	31.7 (684)	39.3	20.0	44.9
	Yes	62.7 (47)	54.8 (85)	68.3 (1475)	63.8	70.6	83.7
Non-HIV comorbidities ever diagnosed with	None	42.7 (32)	30.3 (47)	42.3 (914)	53.1	53.2	72.6
	1 only	29.3 (22)	37.4 (58)	18.1 (390)	45.5	51.7	74.1
	2+	28.0 (21)	32.3 (50)	39.6 (855)	66.7	38.0	68.9
Aware of the number of medicines in their HIV regimen	No	25.3 (19)	21.9 (34)	26.5 (573)	42.1	47.1	62.5
	Yes	74.7 (56)	78.1 (121)	73.5 (1586)	58.9	47.9	74.7

Because of missing data, sum of individual categories for some indicators may not add up to total

*ART* antiretroviral therapy, *HCP* healthcare provider

^a^Other Asian countries were three in number: China, Taiwan, and South Korea

^b^Non-Asian countries were 21 in number: Argentina, Australia, Austria, Belgium, Brazil, Canada, Chile, France, Germany, Italy, Mexico, Netherlands, Poland, Portugal, Ireland, Russia, South Africa, Spain, Switzerland, UK, and USA

^c^Noncollapsed percentages for the Likert-type scale measuring medication satisfaction was as follows: Japan (Very unsatisfied, 1.3%; Unsatisfied, 10.7%; Neither satisfied nor unsatisfied, 33.3%; Satisfied, 46.7%; Very satisfied, 8.0%). Other Asian countries (Very unsatisfied, 3.2%; Unsatisfied, 12.9%; Neither satisfied nor unsatisfied, 36.1%; Satisfied, 40.6%; Very satisfied, 7.1%). Non-Asian countries (Very unsatisfied, 2.8%; Unsatisfied, 5.5%; Neither satisfied nor unsatisfied, 20.2%; Satisfied, 47.5%; Very satisfied, 23.9%)

^d^Estimates suppressed because of small sample size

### Prevalence of ART Satisfaction and Perceived Challenges with Treatment

The percentage reporting ART satisfaction was 54.7%[41/75] in Japan and did not differ significantly from other Asian countries (47.7%[74/155], χ^2^(1) = 0.970, p = 0.325) (Table 1). Significantly lower ART satisfaction was reported by Japanese adults who perceived their HCP did not fully meet their needs/priorities compared to those who perceived their needs were met (39.3% [11/28] vs 63.8% [30/47], χ^2^(1) = 4.265, p = 0.039) as well as those hiding vs not hiding their HIV medication (45.8% [22/48] vs 70.4% [19/27], χ^2^(1) = 4.198, p = 0.040).

**Fig. 2:**
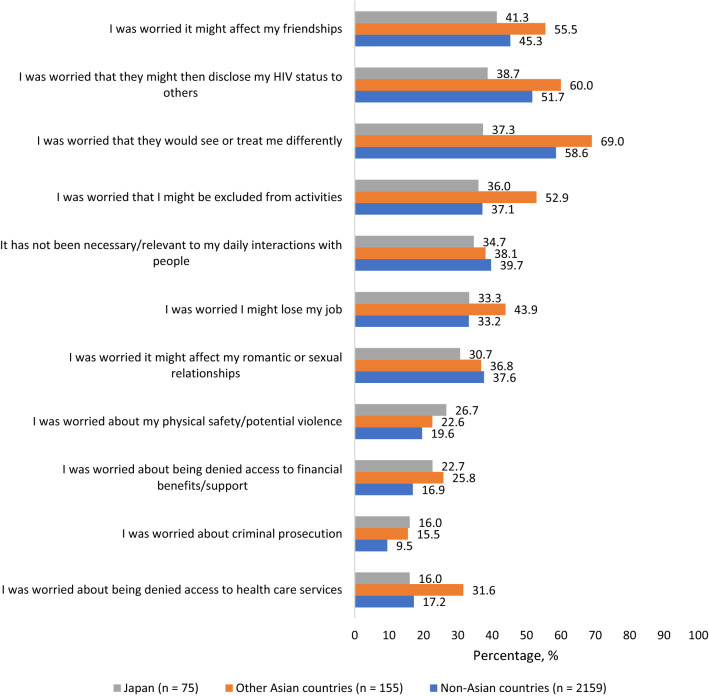
Reasons for refusing to share HIV status with others in the past among people living with HIV in Japan, other Asian countries, and non-Asian countries combined, Positive Perspectives Study, 2019 (Color figure online)

**Fig. 3:**
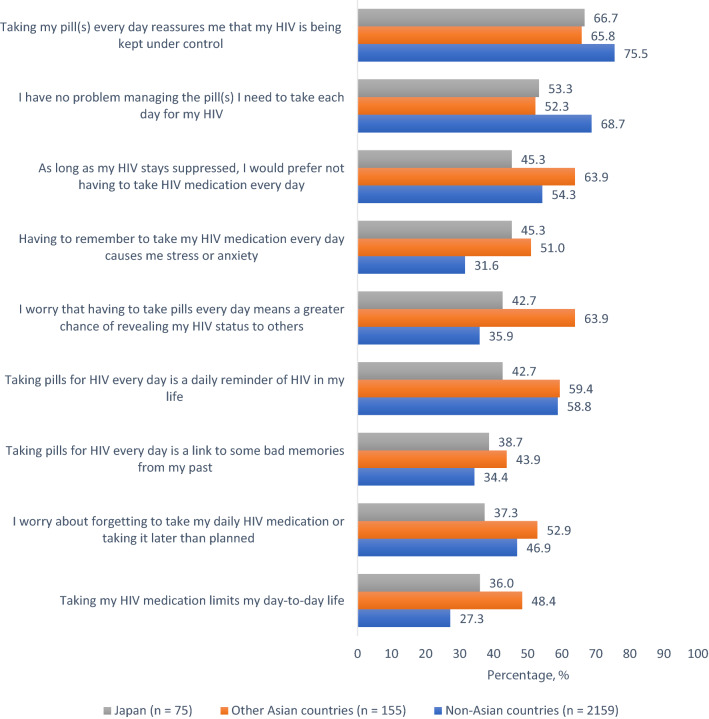
Experiences and attitudes towards current HIV medication among people living with HIV in Japan, other Asian countries, and non-Asian countries combined, Positive Perspectives Study, 2019 (Color figure online)

Privacy concerns among PLHIV in Japan were mostly rooted in fear that disclosure might affect their friendships (41.3%[31/75]), might result in gossips about their HIV status (38.7%[29/75]), might lead to their being treated differently (37.3%[28/75]), or even being excluded from activities (36.0%[27/75]) (Fig. 2). The fear of being treated differently (69.0% vs 37.3%, χ^2^(1) = 20.949, p < 0.001) or being denied access to health services (31.6% vs 16.0%, χ^2^(1) = 6.322, p = 0.012) was almost double in other Asian countries compared to Japan (Fig. 2). The HIV pill was seen by some as an emblem or a trigger of internal and external HIV stigma; for example, 42.7%[32/75] of participants in Japan perceived that daily dosing was a reminder of HIV in their life (Fig. 3). Participants reported a high degree of pill fatigue as well; for example, 45.3%[34/75] of Japanese PLHIV felt stressed by daily oral dosing, consistent with the observation that only 53.3%[40/75] of the same population reported they had no problems with managing their daily oral medications. One-third (36.0%[27/75]) of PLHIV in Japan felt that taking HIV medications daily limited their lives.

**Fig. 4:**
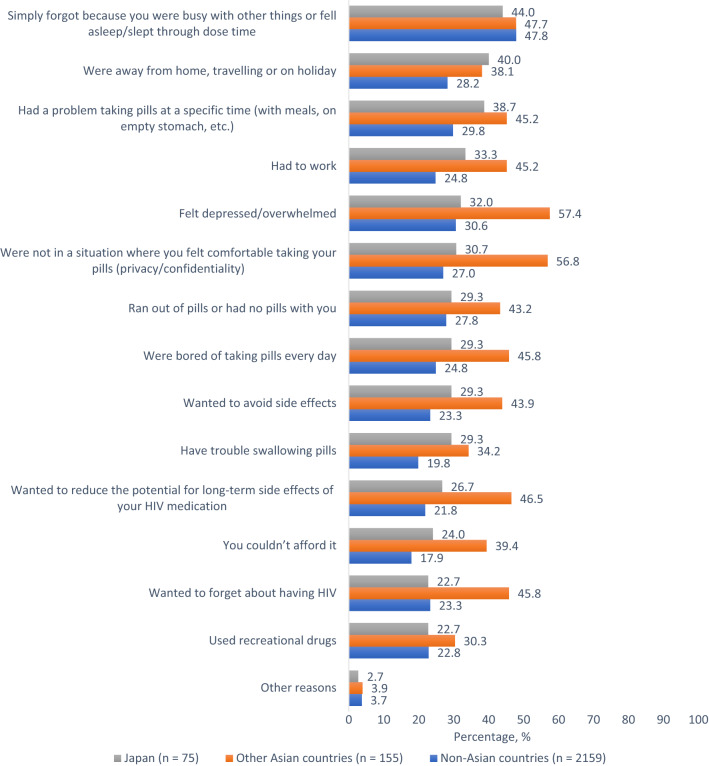
Reasons for missing HIV medications at least once in the past month among people living with HIV in Japan, other Asian countries, and non-Asian countries combined, Positive Perspectives Study, 2019 (Color figure online)

Untoward treatment effects (anticipated or experienced) as well as inconvenient dosing schedules were common reasons for missing ART. For example, in Japan, 22.7%[17/75] missed ART at least once in the past month because they wanted to forget about HIV, 29.3%[22/75] each because of ART side effects and difficulty swallowing, 32.0%[24/75] because they were depressed/overwhelmed, and 38.7%[29/75] because they had problems taking pills at a specific time or with meals. However, the top two reasons for missing ART among participants in Japan were nonmedical reasons: being busy (44.0%[33/75]) and traveling (40.0%[30/75]). The percentage who missed ART at least once in the past month for the following reasons was about twice higher among participants in other Asian countries compared to those in Japan: depressed/ overwhelmed (57.4%[89/115] vs 32.0%[24/75], χ^2^(1) = 13.063, p < 0.001), were in a setting where others could see them (56.8%[88/155] vs 30.7%[23/75], χ^2^(1) = 13.797, p < 0.001), were concerned about long-term side effects (46.5%[72/155] vs 26.7%[20/75], χ^2^(1) = 8.244, p = 0.004) or just because they wanted to forget about HIV (45.8%[71/155] vs 22.7%[17/75], χ^2^(1) = 11.457, p = 0.001, Fig. 4). For all other reasons for missing ART, no significant differences were seen. The percentage who missed ART ≥ 1  time in the past month for any reason was significantly lower in Japan (65.3%[49/75]) vs other Asian countries (83.9%[130/155], χ^2^(1) = 10.065, p = 0.002), but did not differ significantly from non-Asian countries (70.4%[1520/2159], χ^2^(1) = 0.891, p = 0.345).

### Bivariate Analyses of the Relationship Between ART Satisfaction and Health-Related Outcomes

Individuals reporting ART satisfaction had a significantly higher prevalence of self-rated health in the different domains assessed, compared to those not satisfied. In Japan, for example, those reporting ART satisfaction reported higher optimal mental health (61.0%[25/41] vs 29.4[10/34], χ^2^(1) = 7.440, p = 0.006), sexual health (65.9%[27/41] vs 29.4%[10/34], χ^2^(1) = 9.875, p = 0.002), and overall health (65.9%[27/41] vs. 38.2%[13/34], χ^2^(1) = 5.696, p = 0.017). Negative attitudes towards HIV medication were significantly lower among those reporting ART satisfaction than those non-satisfied, positive attitudes in contrast were higher among those satisfied. The percentage in Japan who perceived that HIV medicines were a link to some bad memories from their past was 26.8%[11/41] among those ART-satisfied vs 52.9%[18/34] among those non-satisfied (χ^2^(1) = 5.344, p = 0.021). Conversely, the percentage in Japan reporting they had no problems managing their HIV medication was 68.3%[28/41] vs 35.3%[12/34] respectively (χ^2^(1) = 8.132, p = 0.004); similarly, the percentage perceiving that taking their HIV medication was a reassurance that their viral load was under control was 80.5%[33/41] vs 50.0%[17/34] respectively (χ^2^(1) = 7.774, p = 0.005). Consistent patterns were seen in other Asian countries as well as non-Asian countries (Table 2).

**Table 2:** Percentage of participants who reported various treatment challenges and experiences among people living with HIV in Japan, other Asian countries, and non-Asian countries combined, Positive Perspectives Study, 2019

Indicator	Japan			Other Asian countries^a^			Non-Asian countries^b^		
	Total	ART non-satisfied	ART-satisfied	Total	ART non-satisfied	ART-satisfied	Total	ART non-satisfied	ART-satisfied
	(n = 75)	(n = 34)	(n = 41)	(n = 155)	(n = 81)	(n = 74)	(n = 2159)	(n = 617)	(n = 1542)
Experiences and attitudes towards current HIV medication									
Taking my pill(s) every day reassures me that my HIV is being kept under control	66.7	50.0	80.5	65.8	51.9	81.1	75.5	60.0	81.7
Having to remember to take my HIV medication every day causes me stress or anxiety	45.3	50.0	41.5	51.0	66.7	33.8	31.6	41.7	27.6
Taking my HIV medication limits my day-to-day life	36.0	44.1	29.3	48.4	65.4	29.7	27.3	38.2	22.9
I have no problem managing the pill(s) I need to take each day for my HIV	53.3	35.3	68.3	52.3	37.0	68.9	68.7	51.5	75.6
Taking pills for HIV every day is a daily reminder of HIV in my life	42.7	47.1	39.0	59.4	75.3	41.9	58.8	61.6	57.7
Taking pills for HIV every day is a link to some bad memories from my past	38.7	52.9	26.8	43.9	60.5	25.7	34.4	41.3	31.6
I worry about forgetting to take my daily HIV medication or taking it later than planned	37.3	47.1	29.3	52.9	55.6	50.0	46.9	51.9	44.9
I worry that having to take pills every day means a greater chance of revealing my HIV status to others	42.7	50.0	36.6	63.9	69.1	58.1	35.9	45.9	31.9
As long as my HIV stays suppressed, I would prefer not having to take HIV medication every day	45.3	35.3	53.7	63.9	69.1	58.1	54.3	59.3	52.3
As long as my viral load is suppressed, I am open to taking an HIV treatment composed of fewer medicines	68.0	70.6	65.8	63.9	61.7	66.2	73.0	63.8	76.6
Reasons for missing HIV medications at least once in the past month									
Were away from home, travelling or on holiday	40.0	52.9	29.3	38.1	39.5	36.5	28.2	35.0	25.5
Were not in a situation where you felt comfortable taking your pills (privacy/confidentiality)	30.7	35.3	26.8	56.8	64.2	48.6	27.0	36.6	23.1
Simply forgot because you were busy with other things or fell asleep/slept through dose time	44.0	50.0	39.0	47.7	55.6	39.2	47.8	54.6	45.1
Have trouble swallowing pills	29.3	47.1	14.6	34.2	49.4	17.6	19.8	28.5	16.3
Wanted to avoid side effects	29.3	32.4	26.8	43.9	54.3	32.4	23.3	31.9	19.8
Wanted to reduce the potential for long-term side effects of your HIV medication	26.7	35.3	19.5	46.5	58.0	33.8	21.8	32.1	17.7
Used recreational drugs	22.7	29.4	17.1	30.3	38.3	21.6	22.8	27.9	20.8
Felt depressed/overwhelmed	32.0	44.1	22.0	57.4	63.0	51.4	30.6	41.8	26.1
Were bored of taking pills every day	29.3	38.2	22.0	45.8	66.7	23.0	24.8	35.7	20.5
Wanted to forget about having HIV	22.7	32.4	14.6	45.8	54.3	36.5	23.3	30.3	20.4
Had a problem taking pills at a specific time (e.g., with meals, on empty stomach)	38.7	50.0	29.3	45.2	55.6	33.8	29.8	38.7	26.2
Ran out of pills or had no pills with you	29.3	29.4	29.3	43.2	56.8	28.4	27.8	35.5	24.8
Had to work	33.3	41.2	26.8	45.2	48.1	41.9	24.8	31.8	22.0
You couldn’t afford it	24.0	35.3	14.6	39.4	48.1	29.7	17.9	23.3	15.7
Other reasons	2.7	2.9	2.4	3.9	6.2	1.4	3.7	4.5	3.3
Reasons for refusing to share HIV status with others in the past									
It has not been necessary/relevant to my daily interactions with people	34.7	26.5	41.5	38.1	42.0	33.8	39.7	36.3	41.1
I was worried that they would see or treat me differently	37.3	29.4	43.9	69.0	66.7	71.6	58.6	60.6	57.8
I was worried that they might then disclose my HIV status to others	38.7	41.2	36.6	60.0	55.6	64.9	51.7	54.0	50.8
I was worried that I might be excluded from activities	36.0	29.4	41.5	52.9	51.9	54.1	37.1	41.5	35.4
I was worried about being denied access to health care services	16.0	20.6	12.2	31.6	32.1	31.1	17.2	22.4	15.1
I was worried about being denied access to financial benefits/support	22.7	35.3	12.2	25.8	23.5	28.4	16.9	22.4	14.7
I was worried it might affect my friendships	41.3	38.2	43.9	55.5	50.6	60.8	45.3	48.6	44.0
I was worried I might lose my job	33.3	32.4	34.1	43.9	38.3	50.0	33.2	38.1	31.3
I was worried it might affect my romantic or sexual relationships	30.7	26.5	34.1	36.8	30.9	43.2	37.6	39.5	36.8
I was worried about my physical safety/potential violence	26.7	29.4	24.4	22.6	19.8	25.7	19.6	25.1	17.4
I was worried about criminal prosecution	16.0	23.5	9.8	15.5	18.5	12.2	9.5	11.2	8.8
Self-rated health									
Optimal physical health	52.0	41.2	61.0	41.9	23.5	62.2	61.7	42.5	69.4
Optimal mental health	46.7	29.4	61.0	36.8	24.7	50.0	59.5	42.0	66.5
Optimal sexual health	49.3	29.4	65.9	32.9	18.5	48.6	49.7	36.1	55.2
Optimal overall health	53.3	38.2	65.9	36.8	19.8	55.4	59.3	37.8	67.9

*ART* antiretroviral therapy, *HCP* Healthcare provider

^a^Other Asian countries were three in number: China, Taiwan, and South Korea

^b^Non-Asian countries were 21 in number: Argentina, Australia, Austria, Belgium, Brazil, Canada, Chile, France, Germany, Italy, Mexico, Netherlands, Poland, Portugal, Ireland, Russia, South Africa, Spain, Switzerland, UK, and USA

### SEM of the Relationship Between ART Satisfaction and Health-Related Outcomes

The final SEM fitted the data well as demonstrated by various goodness-of-fit statistics (TLI = 0.945; CFI = 0.957; RMSEA = 0.049) (Fig. 1). High factor loadings were seen on all three latent variables along with high internal consistency (“health status”, Cronbach α = 0.8408; “provider engagement”, α = 0.8357; and “ART anxiety”, α = 0.8966). Factors positively correlated with ART satisfaction were high “provider engagement” (β = 0.35, 95% CI 0.31 to 0.38), high level of perceived control over ART dosing schedule (β = 0.28, 95% CI 0.24 to 0.32), and high level of belief that ART prevents HIV transmission (β = 0.16, 95% CI 0.12 to 0.19). Conversely, an inverse relationship with ART satisfaction was seen for the following: greater experience of side effects (β = − 0.24, 95% CI − 0.28 to − 0.21), high “ART anxiety” (β = − 0.20, 95% CI − 0.24 to − 0.16); and being on multi-tablet regimens (β = − 0.13, 95% CI − 0.17 to − 0.09). Those reporting ART satisfaction reported better “health status” (β = 0.33, 95% CI 0.30 to 0.37) and greater adherence (β = 0.13, 95% CI 0.09 to 0.17). Similarly, the belief that ART prevents HIV transmission increased the likelihood of optimal adherence (β = 0.06, 95% CI 0.02 to 0.10) whereas high level of ART side effects was negatively associated with optimal adherence (β = − 0.10, 95% CI − 0.14 to − 0.06).

**Fig. 5:**
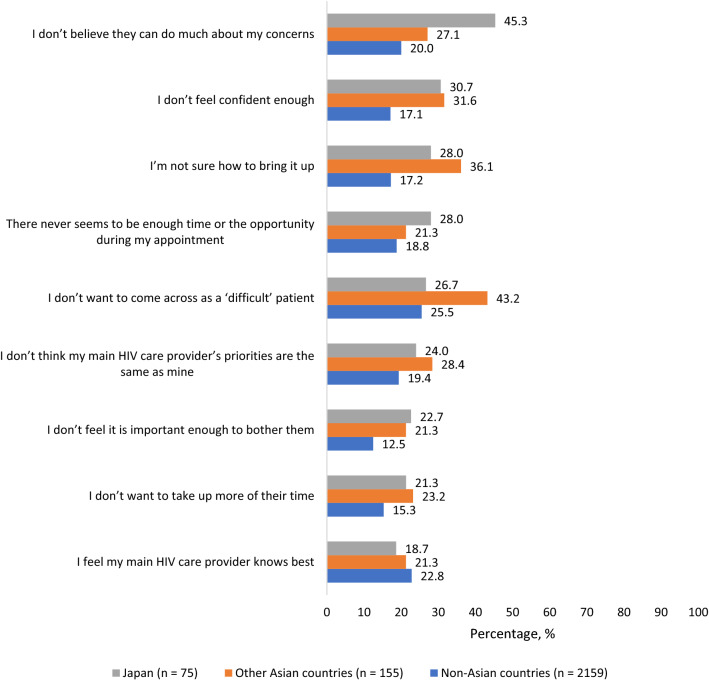
Percentage of participants who reported various barriers to discussing salient health issues with their healthcare provider, Positive Perspectives Study, 2019 (Color figure online)

**Fig. 6:**
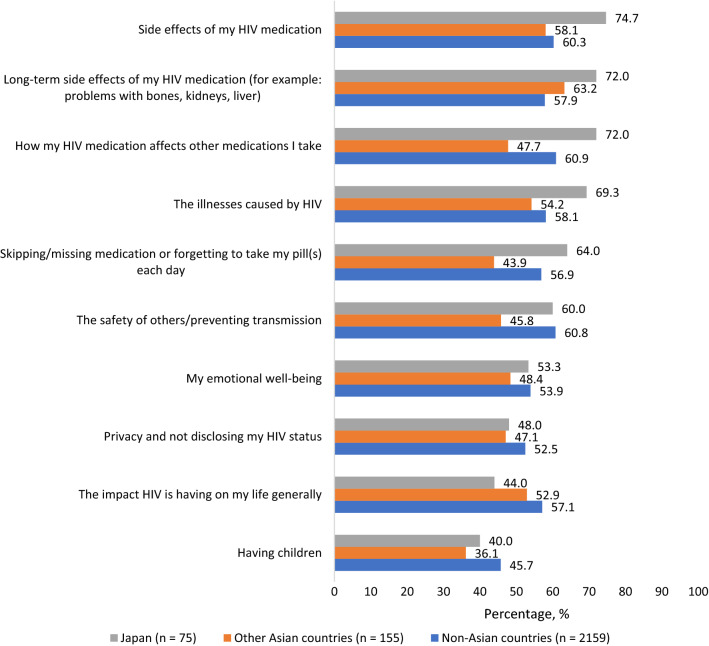
Percentage of participants who felt comfortable discussing specified health-related concerns with their healthcare provider, Positive Perspectives Study, 2019 (Color figure online)

Even though HCP engagement was a significant contributor to treatment satisfaction (Fig. 1), many participants reported barriers to discussing salient issues with their HCPs. For example, participants in Japan reported the highest percentage for those uncomfortable to broach issues with their HCPs out of the belief nothing much could be done to help them (45.3%[34/75] in Japan, vs 27.1%[42/155] in other Asian countries, and 20.0%[432/2159] in non-Asian countries, χ^2^(2) = 31.132, p < 0.001) (Fig. 5). Compared to those in non-Asian countries, participants in Japan were more likely to report they did not feel confident enough to initiate discussions with their HCP (30.7%[23/75] vs 17.1%[370/2159], χ^2^(1) = 9.151, p = 0.002), they were not sure how to bring the issue up (28.0%[21/75] vs 17.2%[372/2159], χ^2^(1) = 5.799, p = 0.016), that there never seemed to be enough time/opportunity during their appointment (28.0%[21/75] vs 18.8%[406/2159], χ^2^(1) = 3.964, p = 0.046), or perceiving that whatever concern they had was not important enough to “bother” their HCP (22.7%[17/75] vs 12.5%[269/2159], χ^2^(1) = 6.765, p = 0.009). The percentage who reported being comfortable discussing an array of specific issues with their HCPs is shown in Fig. 6.

### Maximum Diffusion Experiment of Patient Preferences

**Fig. 7:**
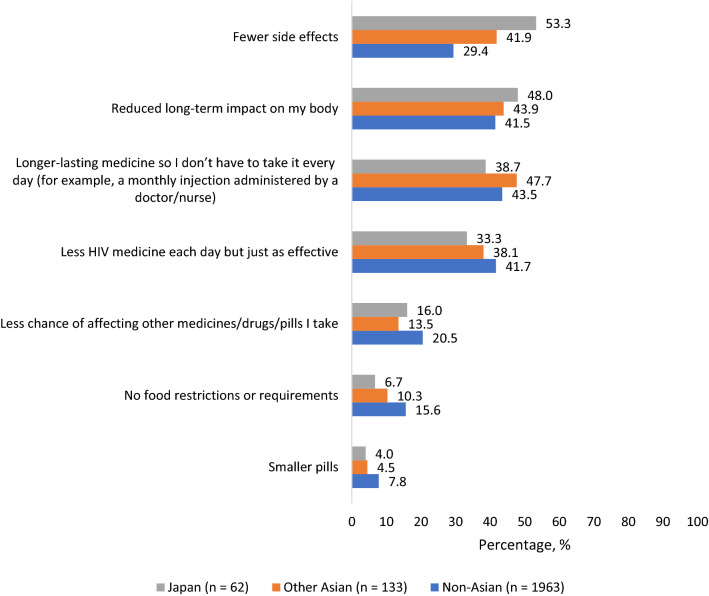
Percentage of participants who ranked each of the listed attributes as the first or second most important treatment improvement of HIV medicines out of seven attributes assessed, Positive Perspectives Study, 2019. Analysis restricted to the subset of participants who completed the maximum diffusion experiment that was embedded as part of the Positive Perspectives survey (Color figure online)

The most preferred ART improvements based on first or second-place rankings by PLHIV in Japan were ART with “fewer side effects” (53.3%), “reduced long-term impact on my body” (48.0%), and “longer-lasting medicine so I don’t have to take it every day” (38.7%). Preference shares for ART with fewer side effects and reduced long-term impact were numerically higher in Japan than in other Asian countries or non-Asian countries (Fig. 7). The percentage ranking nondaily regimens in first or second place in terms of perceived importance was 38.7% in Japan, 47.7% in other Asian countries, and 43.5% in non-Asian countries (Fig. 7); the percentage willing to try nondaily regimens was 45.3% in Japan, 63.9% in other Asian countries and 54.3% in non-Asian countries (Table 2). Similarly, the percentage ranking ART with fewer medicines in first or second place in terms of perceived importance was 33.3% in Japan, 38.1% in other Asian countries, and 41.7% in non-Asian countries, while the percentage willing to try ART with fewer medicines was 68.0% in Japan, 63.9% in other Asian countries and 73.0% in non-Asian countries.

## Discussion

This study provides a comprehensive assessment of factors associated with treatment satisfaction among people living with HIV in Japan and other selected countries. Using a Structural Equation Modeling that examined the complex, and multidirectional relationships between ART satisfaction, medication challenges, patient characteristics, as well as clinical and provider contexts, we found that the strongest positive influences on ART satisfaction were high levels of provider engagement and patient perceived control over their ART dosing schedule, whereas the strongest negative influence on ART satisfaction was medication side effects. Not surprisingly, in ranked choice experiments, the treatment attribute that received the highest share of either the first or second place ranking as the most important improvement to HIV medicines was reducing side effects (53.3% of Japanese participants).

Despite better adherence, PLHIV in Japan reported similar, not higher rates of ART satisfaction, compared to those in other Asian countries, reinforcing the idea that the presence of good clinical indicators (e.g., adherence or viral suppression), may not necessarily translate into good treatment experience, or by extension, good health-related quality of life [22]. Providers therefore must look beyond focusing solely on those objectively measured clinical indicators and incorporate the “voice” of the patient when planning treatment. It is however not enough to wait for patients to spontaneously share information about their ART challenges as this could lead to under-reporting [7, 9], especially in cultures like Japan that value stoicism [12]. Good quality communication, including use of viewpoint questions, may be needed to draw out patients during consultations. Providers can use what they learn during such consultations to positively impact ART satisfaction and adherence by tailoring treatment to address specific concerns patients may have about ART, be they emotional, psychosocial, or medical challenges [7, 9, 23]. HCPs can also provide patients with information on new treatment options to help them make well-informed decisions [7, 24]. Besides virologic control, considering patients’ preferences in relation to quality of life can accelerate progress towards reaching the targets related to improving adherence and quality of life [5]. According to the HIV treatment guideline of the Japanese Ministry of Health, Labor and Welfare [25], achieving virological suppression alone is not enough in the context of long-term medical treatment; attention should be paid to improving the quality of life by reducing side effects and improving the convenience of oral administration. The guideline further emphasizes that the principle underpinning drug change or switching is to improve quality of life and maintain viral suppression while ensuring that future treatment options are not narrowed.

Nonmedical reasons, including travel, were the leading reasons for missing ART among PLHIV in Japan. In a recent international study [24], PLHIV participants revealed that what they liked the most about long-acting HIV regimens was the ease of travel (i.e., not having to carry along pills). In that same study, 84.2% of healthcare providers indicated willingness to offer long-acting regimens because of convenience and lifestyle reasons [24]. Recognizing that the patient and the person are one and the same, and that disruptions in one aspect of their life would most likely affect all aspects of their life, can motivate providers to seek flexible treatment options that fit into the lives of PLHIV. Results from our structural model showed that side effects had the strongest negative impact on ART satisfaction, whereas provider engagement and perceived control over ART dosing schedule had the strongest positive impacts. Reducing side effects was also the most important treatment improvement in the first or second-place priority ranking by PLHIV in Japan. Taken together, these findings indicate that if HCPs empower PLHIV so they avail themselves of treatments that suit their lifestyle (i.e., increased perceived control) and are highly tolerable, then treatment satisfaction may be achieved, and along with it, greater adherence to ART and improved self-rated health as suggested by our study.

The differences observed in this study underscore the need for providers to be culturally sensitive and patient-centered rather than applying broad stereotypes. Policy-wise, the findings underscore the need for surveillance data at national and subnational levels to better inform public health and clinical practice, programs, and policy.

Some limitations exist to this study. First, these are cross-sectional analyses and only associations can be drawn. Second, the data may not be fully representative because of the non-probabilistic sampling. The findings are however consistent with the scientific literature but propose a more comprehensive evaluation framework of treatment satisfaction among PLHIV.

## Conclusion

A significant number of unmet needs remain for PLHIV relating to daily intake of oral medication. Concerns about drug tolerability or side effects constitute the single most important treatment consideration among persons living with HIV in Japan; 29.3% missed ART ≥ 1 time in the past month to avoid side effects. Factors positively associated with ART satisfaction included perceived control over ART dosing schedule, perceived benefits of treatment, and high provider engagement. Conversely, factors inversely associated with ART satisfaction were side effects, being on multi-tablet regimens, and high “ART anxiety”. High level of satisfaction with ART was significantly associated with good self-rated health and treatment adherence. Pre-emptively considering determinants of treatment satisfaction when planning treatment can go a long way in improving the patient’s experience. Treatment options tailored to patient concerns, preferences, and lifestyle, including long-acting ART, may help address unmet needs, improve quality of life, and improve long-term outcomes for PLHIV.
